# The complete mitochondrial genome of the *Heosemys depressa* (Testudines, Geoemydidae)

**DOI:** 10.1080/23802359.2017.1357438

**Published:** 2017-07-26

**Authors:** Yuqin Wang, Xueting Dai, Meng Wang, Liuwang Nie

**Affiliations:** Life Science College, Anhui Normal University, Provincial Key Lab of the Conservation and Exploitation Research of Biological Resources in Anhui, Wuhu, China

**Keywords:** Mitogenome, *Heosemys depressa*, Testudines

## Abstract

The complete mitochondrial genome of *Heosemys depressa* was obtained and characterized in this study. The mitochondrial genome is a circular molecule of 16,773bp in length, and harbours 13 protein-coding genes (PCGs), 2 ribosomal RNA (rRNA) genes, 22 transfer RNA (tRNA) genes, and 1 non-coding D-loop region (control region). Its gene arrangement type is identical to the type of most vertebrate. Phylogenetic analysis suggests that *H. depressa* is closely related to *H. annandalii* than to the other species. Our data provide a useful resource for the phylogenetic studies of genus *Heosemys*.

The Arakan forest turtle (*Heosemys depressa*), which is critically endangered, is endemic to Myanmar and belongs to the genus Heosemys within the family Geoemydidae (Rahman et al. 2015). The specimen of *H. depressa* (code No. 26080148) was obtained from Hefei Wildlife Park, Anhui province in China (31°49′N, 117°13′E) and stored in Anhui Provincial Key Lab of the Conservation and Exploitation Research of Biological Resources from Anhui Normal University. Total genomic DNA was extracted from *H. depressa* muscle tissue using the standard phenol–chloroform protocol (Zhou et al. 2015), and the complete mtDNA of *H. depressa* was amplified and sequenced using 15 primer pairs.

The complete mitochondrial genome sequence of *H. depressa* is 16,773bp in length (GenBank accession No. JQ266017). The circular mitogenome contains 37 genes, including 13 typical protein-coding genes (PCGs), 22 tRNA genes, 2 rRNA genes (*12S rRNA* and *16S rRNA*), and 1 control region (CR). Nine genes of *ND6*, *tRNA^Pro^*, *tRNA^Gln^*, *tRNA^Ala^*, *tRNA^Asn^*, *tRNA^Cys^*, *tRNA^Tyr^*, *tRNA^Ser(UCN)^*, and *tRNA^Glu^* were encoded on the light-strand, and the remaining were encoded on the heavy-strand. The putative origin of light-strand replication (OL)(28bp) is situated between the *tRNA^Asn^* and *tRNA^Cys^* genes of the WANCY tRNA cluster as most vertebrates (Kan et al. 2010; Ren et al. 2016; Yan et al. 2016) .

The non-coding regions of the *H. depressa* mtDNA included the control region and some intergenic spacers. The CR (1262bp) was located between *tRNA^Pro^* and *tRNA^phe^* genes. Two kinds of variable number tandem repeats (VNTR), AATTTAATAT and AT, were identified at the 3'-end of the CR, which repeated 32 and 5 times, respectively.

The 13 identified PCGs were 11388bp in total length (168–1806bp). PCGs began with ATG as start codon except *COI* with GTG. Stop codons were variable for all protein-coding genes, and five genes (*ATP8*, *ATP6*, *ND4L*, *ND4*, *ND5*) used TAA, whereas, *ND1, ND2, ND3, COIII* stopped with TAG, and *COI* and *ND6* ended with AGG. Incomplete stop codons (T– –) were found in *COIII* and *Cyt b*. There was an additional inserted nucleotide A at the position 174 of the *ND3* gene, which had been reported in some other species of Testudines (Parham et al. 2006).

The length of 22 tRNA genes ranged from 66bp (*tRNA^Cys^* and *tRNA^Ser(AGN)^*) to 76bp (*tRNA^Trp^* and *tRNA^Leu(UUR)^*). The two rRNAs were 963bp (*12S*) and 1602bp (*16S*) in length, respectively.

The complete mitogenomes sequences of *H. depressa* and other species belonging to Testudines were used for phylogenetic analysis, with setting *Pelodiscus sinensis* and *Lissemys punctata* as outgroups. Maximum likelihood (ML), Maximum parsimony (MP), and Neighbor joining (NJ) method were used to examine the system evolution status of *H. depressa.* As shown in the phylogenetic tree (Figure 1), *H. depressa* and *H. annandalii,* belonging to genus *Heosemys,* formed a monophyletic group, and the genus *Heosemys* is closely related to genus *Notochelys*, *Cyclemys,* and *Sacalia*. These data promote our understanding of the system evolutionary status of *H. depressa.*

**Figure 1.**
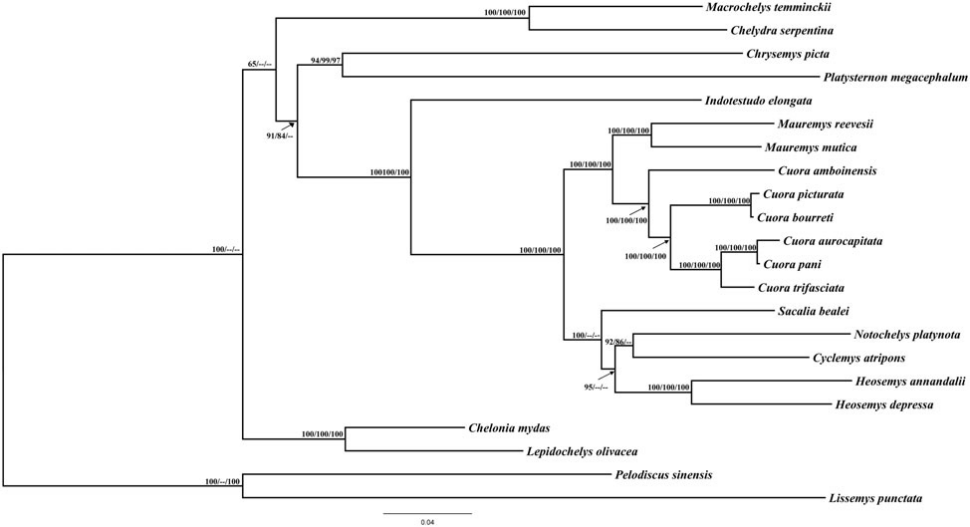
Phylogenetic tree showing the relationship between *H. depressa* and 21 other Testudines species based on the nucleotide dataset of the 13 PCGs, with *Pelodiscus sinensis* and *Lissemys punctata* as outgroups. Numbers above each node indicated the bootstrap support values of ML, MP, and NJ, respectively. Branch lengths and topologies came from the ML analysis. All the GenBank accession numbers of 22 species are listed as below: *Pelodiscus sinensis* NC_006132*, Lissemys punctata* NC_012414*, Chelonia mydas* AB012104*, Lepidochelys olivacea* NC_028634*, Mauremys reevesii* AY676201*, Mauremys mutica* DQ453753*, Cuora trifasciata* NC_022857*, Cuora pani* NC_014401*, Cuora aurocapitata* NC_009509*, Cuora bourreti* NC_017885*, Cuora picturata* NC_017878*, Cuora amboinensis* NC_014769*, Sacalia bealei* GU183364*, Notochelys platynota* NC_020665*, Cyclemys atripons* NC_010970*, Heosemys annandalii* NC_020668*, Heosemys depressa* JQ 266017*, Indotestudo elongate* NC_007695*, Chrysemys picta* NC_002073*, Platysternon megacephalum* DQ 256377*, Chelydra serpentine* NC_011198*, Macrochelys temminckii* EF 071948.
